# Antidyslipidemic Potential of Water-Soluble Polysaccharides of *Ganoderma applanatum* in MACAPOS-2-Induced Obese Rats

**DOI:** 10.1155/2021/2452057

**Published:** 2021-08-18

**Authors:** Adamou Mfopa, Francine K. Mediesse, Clémence Mvongo, Sandrine Nkoubatchoundjwen, Ambe A. Lum, Eugène Sobngwi, René Kamgang, Thaddée Boudjeko

**Affiliations:** ^1^Laboratory of Phytoprotection and Valorization of Genetic Resources, Biotechnology Centre-Nkolbisson, P.O. Box 17673 Etetak, Yaoundé, Cameroon; ^2^Institute of Medical Research and Medicinal Plants Studies (IMPM), P.O. Box. 13033, Yaoundé, Cameroon; ^3^Department of Life Science, Higher Teacher Training College Bertoua, University of Ngaoundéré, Ngaoundéré, Cameroon; ^4^Department of Animal Biology and Physiology, Faculty of Science, University of Yaoundé I, P.O. Box 812, Yaoundé, Cameroon; ^5^Laboratory of Metabolic Diseases and Metabolism, Biotechnology Centre-Nkolbisson, P.O. Box 17673 Etetak, Yaoundé, Cameroon; ^6^Department of Biochemistry, Faculty of Science, University of Yaoundé I, P.O. Box 812, Yaoundé, Cameroon

## Abstract

Increased consumption of high-calorie foods leads to obesity usually associated with metabolic disorders including diabetes, hyperglycemia, and dyslipidemia. *Ganoderma applanatum* is a nonedible mushroom traditionally used in West Cameroon for the treatment of many diseases including hypertension, diabetes, and hepatitis. This study was designed to investigate the antidyslipidemic potential of water-soluble polysaccharides of *G. applanatum* in MACAPOS-2- (maize, cassava, palm oil, and sugar) induced obese rats. For this purpose, obesity was induced on 6–8-week-old male Wistar rats with a local high-fat diet for four months. *G. applanatum* polysaccharides (GAPs) obtained by hot water extraction were orally administered to obese rats for two months at different dose levels (50, 100, and 150 mg/kg bodyweight), and its potential was investigated on food consumption, bodyweight gain, serum, and tissue lipids parameters. GAP extract increased the bodyweight gain by raising the food intake of obese rats. Furthermore, the administration of GAP extract at different dose levels significantly decreased the total cholesterol, triglyceride, low-density lipoprotein cholesterol levels, and the atherogenic index from 50 to 150 mg/kg bodyweight. Conversely, GAP extract improved the high-density lipoprotein cholesterol level in obese rats compared with untreated rats after two months' study period. These results indicated that GAP extract may be considered as a novel bioactive compound against dyslipidemia and its associated complications.

## 1. Introduction

Obesity, one of the most serious public health challenges of the 21^st^ century, is generally due to overconsumption of energy-dense food, mainly a high-fat diet. Among the 7.5 billion of the world population, 2 billion were found to be overweight and 650 million obese. The worldwide prevalence of obesity nearly tripled in the past decades, thus becoming pandemic [1]. Obesity is linked to various comorbidities, driving mainly by its associated metabolic disorders leading to type 2 diabetes, impaired glucose tolerance, hypertension, and dyslipidemia [2–4]. The pathogenesis of obesity is complex and not entirely understood but involves the interactions of numerous genetic, dietary, lifestyle, and environmental factors [5]. A sedentary lifestyle and consumption of a saturated fatty acid-rich and/or sugar-rich diet are predispositions for obesity. Management of obesity and associated disorders remains a complicated medical challenge. Many treatments have been actively developed, but have not fulfilled all the expectations due to the lack of efficacy or the induction of deleterious side effects [6] as cardiovascular disorders, steatorrheas. Thus, there is a great interest in medicinal plants which are available and are sources of biologically active components.

Polysaccharides have attracted more and more attention in the pharmaceutical sector due to their no or less toxicity, their availability from widespread sources, and their broad spectrum of biological activities. Most of mushroom polysaccharides are *β*-glucan polymers, with the main chain consisting of *β*-(1 ⟶ 3) linkages with some *β*-(1 ⟶ 6) branches, but some are true heteroglycans [7]. Several of them display antidyslipidemic activities [8–11]. Contrary to higher plants where numerous research studies are performed, mushrooms comprised a vast and yet largely untapped source of powerful new pharmaceutical products. They represented a broad source of polysaccharides with antitumor and immunostimulating properties in particular and, most importantly, for modern medicine. Mushroom's polysaccharides are capable of modulating the immune response in animals and humans and inhibiting the growth of certain tumours [12, 13]. Many basidiomycetes mushrooms, if not all, contain biologically active polysaccharides in fruiting bodies, cultured mycelium, and culture broth.

*Ganoderma* is a genus of polypore mushrooms that grow in decaying logs or tree stumps [14]. The fruit body of *Ganoderma*, for its perceived benefits, has gained wide popular use as a dietary supplement in Cameroon and others regions of the world including China, Japan, and North America [15]. *Ganoderma* species are also used in folk medicine to cure various diseases, and strains are commercially cultivated for the preparation of health tablets or drinks. As a kind of health food, it has also been used to prevent and treat immunological diseases as hypertension and tumorigenesis [16, 17]. The beneficial health properties of *Ganoderma* species are attributed to a wide variety of bioactive components, such as polysaccharides, triterpenes, sterols, lectins, and other proteins [18, 19]. Various kinds of bioactive polysaccharides have been isolated from the fruiting bodies of different *Ganoderma* species [20–24]. They represent a structurally diverse class of biological macromolecules with a large spectrum of physiological properties. *G. applanatum* is a nonedible mushroom with an unvarnished, wrinkled, and a white underside surface [25]. It is known to indigenous tribes in West Cameroon as having certain medicinal properties (treatment of headaches, hypertension, tiredness, diabetes, and hepatitis), though still undocumented. This study was designed to investigate the antidyslipidemic potential of water-soluble polysaccharides of *G*. *applanatum* in MACAPOS-2-induced obese rats.

## 2. Materials and Methods

### 2.1. Extraction of Water-Soluble Polysaccharides

Fresh fruiting bodies of *G. applanatum* were collected from Santa (northwest region of Cameroon) in October 2016 and identified by Dr. Rosemary Tonjock Kinge of the Department of Biological Sciences, Faculty of Science of the University of Bamenda, and transferred to the laboratory at the Biotechnology Centre of Nkolbisson in Yaoundé by vehicle. They were shade dried at room temperature for four weeks, then cut into small pieces, and powdered with a grinder. Water-soluble polysaccharides of *G. applanatum* were prepared according to the method described by Liu et al. [26] with slight modifications. Briefly, 250 g of previously obtained powder were boiled with 5 L of distilled water at 100°C for 3 h. The mixture was filtered, and the supernatant was collected and precipitated at 4°C for 24 h with 95° ethanol (1 : 3 (v/v)). The polysaccharides were collected by centrifugation (4000 g; 10 min), then dissolved in distilled water, and lyophilized. Water-soluble polysaccharides powder of *G. applanatum* named GAPs obtained (5.35 g, yield: 2.14%) was stored at 4°C for further use.

### 2.2. Experimental Animals

Male albino Wistar rats (6–8 weeks old), weighing 80–100 g, were raised in a pathogen-free environment under normal environmental conditions in the animal house of the Laboratory of Endocrinology and Radioisotopes of the Institute of Medical Research and Medicinal Plants Studies (IMPM), Yaoundé, Cameroon. The animals were kept in polypropylene cages with a metal mesh cover to acclimatize at an ambient temperature of 20 ± 3°C, 12 hours' light/dark cycle, and adequate ventilation. Then, food and tap water were given *ad libitum*. The experiments were carried out during the light period (9.00–16.00 h). Animal handling and experiments were performed according to the European Union directives on ethical evaluation of animal experiments [27] adopted by the Cameroon Institutional National Ethics Committee, Ministry of Scientific Research and Innovation.

### 2.3. Induction of Obesity

Obesity was induced according to the method described by Kamgang et al. [28]. In brief, after the acclimatization period during two weeks in the animal house, male albino Wistar rats were randomly submitted to normal diet (ND: carbohydrate 50–55%, fat 15–20%, and protein 25–30%) and high-fat diet (HFD: carbohydrate 35–40%, fat 50–55%, and protein 10–15%) (Table 1). Food and tap water intake were measured every two days and animal's weight taken weekly throughout four months of induction. At the end of the induction period, the nose-to-anus length was measured, then the weight was taken, and Lee indexes between groups were calculated in order to select the obese rats. The Lee index was calculated by dividing the cube root of bodyweight (*g*) by the nose-to-anus length (cm) [29].

### 2.4. Experimental Design and Animal Treatment

Animals were weighed, marked for individual identification, divided into various groups, and treated once daily for two months as described in the following:

- Group I (normal control group: NC) consisted of normal rats, allowed free access to a normal diet, and received distilled water
- Group II (obese control group: OBC) consisted of obese rats, allowed free access to an HFD, and received distilled water
- Group III (positive control group: ORC) consisted of obese rats, allowed free access to an HFD, and received orally the reference drug orlistat (F. Hoffmann-La Roche SA) at 20 mg/kg bodyweight
- Group IV to VI (*G. applanatum* polysaccharide-treated groups: GAPs) consisted of obese rats, allowed free access to an HFD, and received GAP extract, respectively, at 50, 100, and 150 mg/kg bodyweight, extrapolated doses from the traditional practitioner

### 2.5. Food and Water Intake during the Treatment

Net food consumption and water intake of each group were calculated every two days as the difference between the quantity of food/water given to animals and that left after a two days' period. Water was provided with graduated drinking bottles. Spilled food pellets were carefully collected to ensure the accuracy of food intake measurements.

### 2.6. Bodyweight during the Treatment

Bodyweight of an individual rat in each group was measured weekly during the entire period of treatment using an electronic weighing balance (Electronic compact scale SF-400 A). Rats were weighed at the beginning of the experiment, and bodyweight gain of rats was calculated as the difference between the final and initial weights and results expressed as a percentage of the initial value.

### 2.7. Collection of the Serum, Liver, and Fat

After two months of treatment, the 12 h overnight-fasted rats were sacrificed by decapitation under ether anesthesia. The blood was collected, allowed to stand for at least 30 min at room temperature, and then, centrifuged at 2500 g for 15 min. After dissection under aseptic conditions, adipose tissues (visceral, subcutaneous, renal, and testicular fats) and the liver were collected and weighed. The liver was immediately washed with an ice-cold saline solution (NaCl 0.9%) and weighed, and 200 mg was homogenized in 1 mL of a Tris-HCl (0.2 M, pH 7.4) buffer solution. The homogenates were centrifuged at 2500 g for 25 min, and the supernatants were collected and stored at −20°C for biochemical analysis.

### 2.8. Determination of Lipid Parameter Content

Total cholesterol, triglyceride, HDL cholesterol, and LDL cholesterol were assayed by enzymatic methods.

The atherogenic index was calculated by dividing total cholesterol by HDL cholesterol (atherogenic index = total cholesterol/HDL cholesterol) [30].

### 2.9. Statistical Analysis

The results were expressed as Mean ± Standard Error of Means. Multigroup comparison was performed by one-way analysis of variance, followed by Dunnett's multiple comparisons as a post hoc analysis test for comparison between groups at *P* < 0.05. Calculations were performed using Graph Pad Prism 7.00 Instat Software.

## 3. Results

### 3.1. Effects of GAP Extract on Food and Water Intake

**Figure 1:**
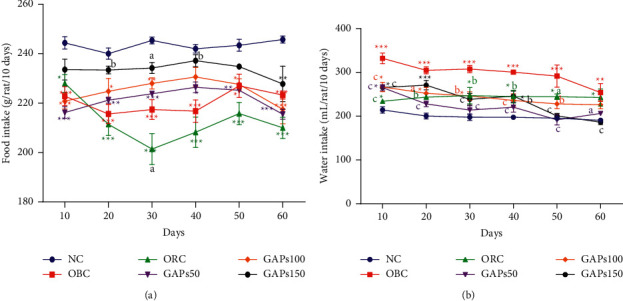
Food intake (a) and water intake (b) of rats after two months of treatment. NC: normal control, OBC: obese control, ORC: orlistat control, obese rats treated with GAPs extract 50 mg/kg (GAPs50), 100 mg/kg (GAPs100), and 150 mg/kg (GAPs150). Significant difference: ^*∗*^*P* <  0.05, ^*∗∗*^*P* < 0.01, ^*∗∗∗*^*P* < 0.001 compared to NC and ^a^*P* < 0.05, ^c^*P* < 0.001 compared to OBC, *n* = 5.

During the treatment, food intake of all GAP-treated groups at 50, 100, and 150 mg/kg increased from 221.20 ± 1.16, 224.80 ± 5.18, and 233.40 ± 1.54 g/rat/10 days at the 20^th^ day to 225.20 ± 0.58, 227.60 ± 2.25, and 234.80 ± 1.07 g/rat/10 days, respectively, at the 50^th^ day as compared to obese control. Afterwards, food intake of normal control rats remained significantly (*P* < 0.001) high during the treatment as compared to obese control (Figure 1(a)). Otherwise, the results showed that oral administration of GAP extract reduced the water intake of rats as compared to obese control. GAPs50, GAPs100, and GAPs150 decreased the water intake from 265.20 ± 7.00, 266.60 ± 10.68, and 265.60 ± 4.48 mL/rat/10 days at the 10^th^ day to 191.60 ± 11.79, 228.00 ± 10.86, and 200.60 ± 5.75 mL/rat/10 days, respectively, at the 50^th^ day. Furthermore, water intake of the normal control rats remained remarkably (*P* < 0.001) low during the treatment (Figure 1(b)).

### 3.2. Effects of GAP Extract on Bodyweight Gain

**Figure 2:**
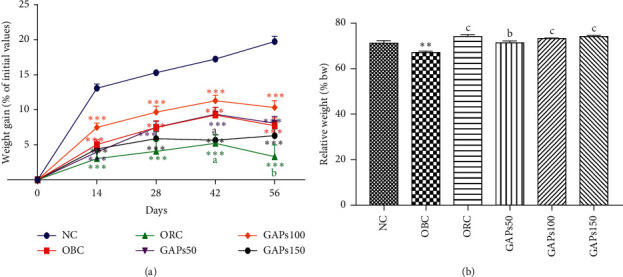
Weight gain (a) and carcass (b) of rats after two months of treatment. NC: normal control, OBC: obese control, ORC: orlistat control, obese rats treated with GAPs extract 50 mg/kg (GAPs50), 100 mg/kg (GAPs100), and 150 mg/kg (GAPs150). Significant difference: ^*∗*^*P* < 0.05, ^*∗∗*^*P* < 0.01, ^*∗∗∗*^*P* < 0.001 compared to NC and ^a^*P* < 0.05, ^c^*P* < 0.001 compared to OBC, *n* = 5.

After two months of treatment, obese control bodyweight gain percentage (7.74%) was significantly (*P* < 0.001) low as compared to normal control (19.76%). However, when obese rats were given GAP extract at the different doses of 50, 100, and 150 mg/kg, their bodyweight gain percentage was 8.09, 10.32, and 6.31%, respectively, at the end of the study period. It appeared that the lowest gain in bodyweight was observed in GAPs150 from 370.60 ± 3.09 g at the first day to 394.00 ± 5.22 g at the 56^th^ day (Figure 2(a)). Moreover, OBC weight's carcass significantly (*P* < 0.01) decreased as compared to NC rats at the end of the study period. The treatment remarkably (*P* < 0.001; *P* < 0.01) increased the weight's carcass of animals: GAPs50 (6.38%), GAPs100 (9.46%), GAPs150 (10.53%), and orlistat (10.62%) as compared to OBC (Figure 2(b)).

### 3.3. Effects of GAP Extract on Adipose Tissue Development

**Figure 3:**
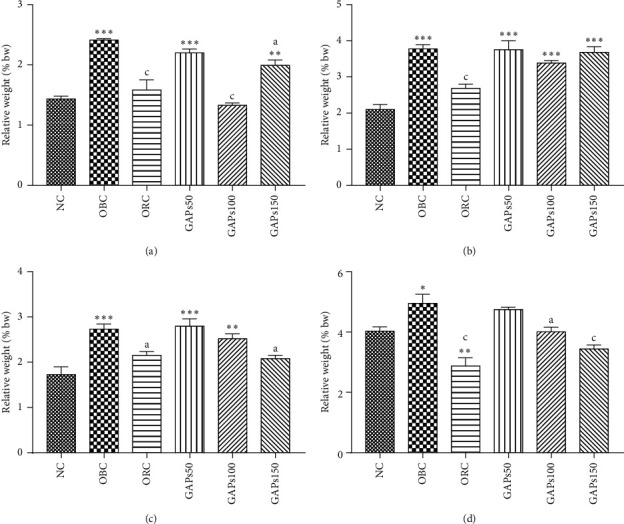
Visceral (a), renal (b), testicular (c), and subcutaneous (d) fat of rats after two months of treatment. NC: normal control, OBC: obese control, ORC: orlistat control, obese rats treated with GAPs extract 50 mg/kg (GAPs50), 100 mg/kg (GAPs100), and 150 mg/kg (GAPs150). Significant difference: ^*∗*^*P* < 0.05, ^*∗∗*^*P* < 0.01, ^*∗∗∗*^*P* < 0.001 compared to NC and ^a^*P* < 0.05, ^c^*P* < 0.001 compared to OBC, *P* = 5.

High-fat diet significantly (*P* < 0.001) increased animals' visceral fat (68.41%), renal fat (79.54%), and testicular fat (58.15%) as compared to normal rats (Figure 3(a)–3(c)). GAPs extract treatment decreased visceral fat by −8.92, −44.92, and −17.38%, respectively, at 50, 100, and 150 mg/kg (Figure 3(a)). Moreover, the effect of this extract on visceral fat was remarkably (*P* < 0.001) at 100 mg/kg bodyweight where the value (1.33%) was below that of the normal control (1.43%). GAP extract treatment also decreased renal fat, respectively, by −0.64, −10.40, and −2.47% at 50, 100, and 150 mg/kg bodyweight (Figure 3(b)). However, concerning testicular fat, the decrease of testicular fat was significant (*P* < 0.05) in rats treated with GAP extract at 150 mg/kg bodyweight (Figure 3(c)). Otherwise, subcutaneous fat of obese rats significantly (*P* < 0.05) increased (22.85%) as compared to normal rats. GAP extract at 50, 100, and 150 decreased subcutaneous fat by −4.07, −18.99, and −30.58%, respectively, as compared to obese control. Subcutaneous fat of GAP-extract-treated groups was comparable to that of the normal animals at the end of the two months' treatment (Figure 3(d)).

### 3.4. Effects of GAP Extract on Serum Lipid Profile

**Figure 4:**
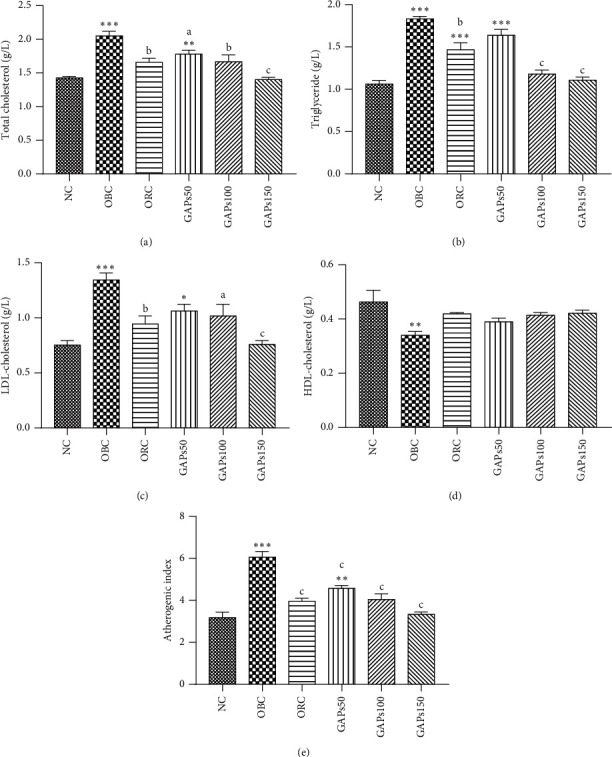
Serum lipid: total cholesterol (a), triglyceride (b), LDL cholesterol (c), HDL cholesterol (d), and atherogenic index (e) of rats after two months of treatment. NC: normal control, OBC: obese control, ORC: orlistat control, obese rats treated with GAPs extract 50 mg/kg (GAPs50), 100 mg/kg (GAPs100), and 150 mg/kg (GAPs150). Significant difference: ^*∗*^*P* < 0.05, ^*∗∗*^*P* < 0.01, ^*∗∗∗*^*P* < 0.001 compared to NC and _a_*P* < 0.05, _c_*P* < 0.001 compared to OBC, *P* = 5.

At the end of two months' treatment, the OBC group had the highest value (*P* < 0.001) of the total cholesterol (2.05 ± 0.07 g/L), triglyceride (1.83 ± 0.03 g/L), and LDL cholesterol (1.35 ± 0.06 g/L) as compared to normal control rats (1.06 ± 0.04 g/L, 1.43 ± 0.02 g/L, and 0.75 ± 0.04 g/L, respectively) (Figure 4(a)–4(c)). The GAP extract at 50, 100, and 150 mg/kg significantly (*P* < 0.05, *P* < 0.01, and *P* < 0.001) decreased the total cholesterol by −13.20, −18.68, and −31.64%, respectively, as compared to obese control (Figure 4(a)). In addition, GAP extract also significantly decreased the triglyceride level (by −10.61, −35.57, and −39.56%) and LDL cholesterol level (by −20.95, −24.32, and −43.52%) at 50, 100, and 150 mg/kg, respectively, after two months' study period as compared to obese control (Figure 4(b) and 4(c)). However, the lowest value of HDL cholesterol was obtained with obese control (0.34 ± 0.01 g/L) while this increased in GAP-extract-treated groups with 0.39 ± 0.01, 0.41 ± 0.01, and 0.42 ± 0.01 g/L, respectively, at 50, 100, and 150 mg/kg (Figure 4(d)). Otherwise, the highest atherogenic index value was obtained in obese control (6.06 ± 0.27). GAPs150 showed the lowest atherogenic index (3.34 ± 0.11) followed by GAPs100 (4.04 ± 0.28) and GAPs50 (4.58 ± 0.14) (Figure 4(e)).

### 3.5. Effects of GAP Extract on the Hepatic Lipid Profile

**Figure 5:**
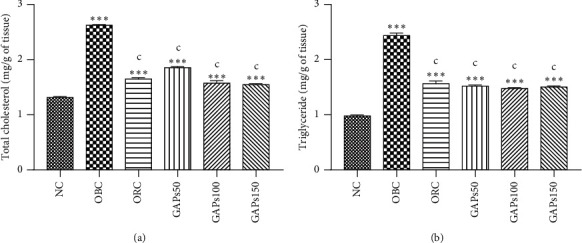
Hepatic lipid profile: total cholesterol (a) and triglyceride (b) of rats after two months of treatment. NC: normal control, OBC: obese control, and ORC: orlistat control; obese rats were treated with GAP extract 50 mg/kg (GAPs50), 100 mg/kg (GAPs100), and 150 mg/kg (GAPs150). Significant difference: ^*∗*^, ^*∗*^, ^*∗*^, *P* < 0.001 compared to NC and *P* < 0.001 compared to OBC, *n* = 5.

High-fat diet increased visibly (*P* < 0.001) hepatic lipids including the total cholesterol and triglyceride. Obese control had the total cholesterol and triglyceride values of about 2.63 ± 0.01 and 2.44 ± 0.05 mg/g of tissue, respectively, compared to normal rats whose total cholesterol and triglyceride values were, respectively, 1.32 ± 0.02 and 0.98 ± 0.02 mg/g of tissue. The GAP extract as well as orlistat significantly (*P* < 0.001) lowered the hepatic total cholesterol and triglyceride levels: GAPs50 (−29.27% and −37.66%, respectively), GAPs100 (−40.07% and −39.44%, respectively), GAPs150 (−41.05% and −38.28%, respectively), and orlistat (−37.13% and −35.78%, respectively). Despite a significant decrease in hepatic lipids observed with treated animals, the proportions of the hepatic total cholesterol and triglycerides remained significantly higher (*P* < 0.001) compared to normal control (NC) animals (Figure 5(a) and 5(b)).

## 4. Discussion

The present research was carried out to investigate the antidyslipidemic potential of water-soluble polysaccharides extract of *G. applanatum* in MACAPOS-2-induced obese rats. Previous studies showed that this local high-fat diet could induce obesity, hyperglycemia associated to glucose intolerance (possibility of insulin resistance), and dyslipidemia by modifying lipid and glucide metabolism probably by altering the action of insulin [28, 31]. Obesity, especially visceral, or central obesity, mainly found in male individuals [32], is known as the main cause of the metabolic syndrome, which includes insulin resistance, type 2 diabetes, hypertension, nonalcoholic fatty liver disease, and dyslipidemia, all risk factors for cardiovascular complications [33, 34]. The typical dyslipidemia of obesity consists of increased fasting and postprandial triglycerides combined with decreased HDL cholesterol and the preponderance of small dense LDL cholesterol. The concentrations of plasma apolipoprotein B are also often increased, partly due to the hepatic overproduction of apolipoprotein-B-containing lipoproteins [35, 36].

Concerning food intake, GAP extract showed a significant effect; increasing food consumption, thus, was effective in increasing the appetite which characterized an orexigenic effect of GAPs. This action could be related to the weight gain observed at the end of the treatment. Previous studies showed the orexigenic actions of crude polysaccharides extract in diabetes mellitus mice [37, 38]. Indeed, overeating can impair hippocampal function. In animals, damages or inactivation of the hippocampus lead to an increase in food intake and reduce the intervals between meals [39, 40]. We also demonstrated in this study that GAPs consumption alleviates the effects of high-fat diet on the adipose tissue development. GAP extract is capable of inducing lipolysis and inhibiting adipocyte differentiation, thus increasing the carcass of animals. For example, Kanagasabapathy et al. [41] showed that *β*-glucans from the *Pleurotus sajor-caju* mushroom prevent the development of obesity and oxidative stress in mice fed a high-fat diet. This antiobesity effect of GAPs is associated with a reduction of lipid storage capacity in the adipose tissue mainly related to a decrease in circulating fatty acids uptake via the lipoprotein lipase expression.

Obesity is associated with rise in triglyceride, LDL cholesterol, total cholesterol, and decreased HDL cholesterol and, thus, is also a risk factor of cardiovascular disease [42]. The atherogenic index was calculated based on the measurement obtained from the serum lipid analysis. The decrease in the atherogenic index in GAP-treated rats indicates a decreased risk of cardiovascular disease [43] for those animals. Another major ﬁnding in our study is that GAP extract lowered the total cholesterol, triglyceride, and LDL cholesterol levels with rise in the HDL cholesterol level. The polysaccharide *β*-glucan has been shown to decrease LDL cholesterol and increase HDL-cholesterol, then to alleviate possibly dyslipidemia, and reduce cardiovascular disease [44]. Similar serum cholesterol-lowering activity was also observed in Maitake, Shiitake, and Enokitake mushrooms [45]. These findings suggested that GAPs had the potential to improve dyslipidemia associated to obesity. Thus, GAPs could be used as a functional food additive to prevent dyslipidemia or as a therapeutic agent to ameliorate symptoms of dyslipidemia. Indeed, GAPs can bind with lipids and act as carriers to participate in the metabolism of cholesterol to accelerate the transport and excretion of serum lipids [8]. Chen et al. [11] found that polysaccharides of the *Pleurotus eryngii* are capable of lowering the level of blood lipids in mice fed a high-fat diet. Similar conclusions were acquired in previous reports [46, 47]. However, more characterization of GAPs is needed to understand how components of the GAP extract could interact with lipids. Moreover, significant decreases in hepatic lipid levels including the total cholesterol and triglyceride in GAP-extract-treated groups testify that GAPs could fight against liver injury. It can be suggested that GAPs are capable of preventing liver steatosis. Indeed, excessive accumulation of adipose tissue caused by an energy imbalance in obesity limits the storage capacity of lipids in adipose tissue and increases the accumulation of fat in other tissues, including the liver, muscle, and heart [33]. This ectopic fat can increase the risk of insulin resistance, type 2 diabetes, and cardiovascular diseases [48].

## 5. Conclusions

The results obtained at the end of this study demonstrated the antidyslipidemic potential of GAP extract in MACAPOS-2-induced obese rat model. The potent antidyslipidemic property of GAPs may result from the reduction of the serum lipid proﬁle. Therefore, our study emphasizes the practical application of GAPs as an external supplement to cope with high-fat-diet-induced adverse effects. Our findings in the integrative medicine field provide a potential track to use GAPs not only as a functional food additive but also as a therapeutic agent to ameliorate symptoms of dyslipidemia. Furthermore, more studies are necessary for further characterization of the bioactive components of GAPs and their structures in order to understand the mechanisms of the antidyslipidemic effects of GAPs in rats.

## Figures and Tables

**Table 1:** Diet composition and their nutritional values.

Diet composition	Normal diet (ND)	High-fat diet (HFD)
	Nutritional values	
Maize	250	80
Wheat	400	110
Soya bean	150	280
Fish flour	100	30
Sucrose	—	50
Palm oil	—	200
Bone flour	10	20
Cabbage palm	80	—
Steeped cassava	—	220
Vitamin complex	10	10
Energy (kcal/Kg)	3400	4730

Components obtained from Yaoundé local market (Cameroon) are expressed in g/kg of diet.

## Data Availability

The data used to support the findings of this study are included within the article.
